# Isofraxidin: Synthesis, Biosynthesis, Isolation, Pharmacokinetic and Pharmacological Properties

**DOI:** 10.3390/molecules25092040

**Published:** 2020-04-27

**Authors:** Mohammad Bagher Majnooni, Sajad Fakhri, Yalda Shokoohinia, Mahdi Mojarrab, Sara Kazemi-Afrakoti, Mohammad Hosein Farzaei

**Affiliations:** 1 Student Research Committee, Kermanshah University of Medical Sciences, Kermanshah 6714415153, Iran; mb.majnooni64@yahoo.com (M.B.M.); sr.kzm73@gmail.com (S.K.-A.); 2 Pharmaceutical Sciences Research Center, Health Institute, Kermanshah University of Medical Sciences, Kermanshah 6734667149, Iran; pharmacy.sajad@yahoo.com (S.F.); yshokoohinia@gmail.com (Y.S.); mmojarrab@kums.ac.ir (M.M.); 3 Ric Scalzo Botanical Research Institute, Southwest College of Naturopathic Medicine, Tempe, AZ 85282, USA

**Keywords:** isofraxidin, biosynthesis, pharmacokinetics, pharmacology, biological activities, hydroxy coumarin

## Abstract

Isofraxidin (7-hydroxy-6, 8-dimethoxy coumarin) (IF) is a hydroxy coumarin with several biological and pharmacological activities. The plant kingdom is of the most prominent sources of IF, which, among them, *Eleutherococcus* and *Fraxinus* are the well-known genera in which IF could be isolated/extracted from their species. Considering the complex pathophysiological mechanisms behind some diseases (e.g., cancer, neurodegenerative diseases, and heart diseases), introducing IF as a potent multi-target agent, which possesses several herbal sources and the multiple methods for isolation/purification/synthesis, along with the unique pharmacokinetic profile and low levels of side effects, could be of great importance. Accordingly, a comprehensive review was done without time limitations until February 2020. IF extraction methods include microwave, mechanochemical, and ultrasound, along with other conventional methods in the presence of semi-polar solvents such as ethyl acetate (EtOAc). In addition to the isolation methods, related synthesis protocols of IF is also of great importance. From the synthesis point of view, benzaldehyde derivatives are widely used as precursors for IF synthesis. Along with the methods of isolation and biosynthesis, IF pharmacokinetic studies showed hopeful in vivo results of its rapid absorption after oral uses, leading to different pharmacological effects. In this regard, IF targets varieties of inflammatory mediators including nuclear factor kappa-light-chain-enhancer of activated B cells (NF-κB), tumor necrosis factor-α (TNF-α), and matrix metalloproteinases (MMPs). thereby indicating anticancer, cardioprotective, and neuroprotective effects. This is the first review on the synthesis, biosynthesis, isolation, and pharmacokinetic and pharmacological properties of IF in combating different diseases.

## 1. Introduction

**Figure 1:**
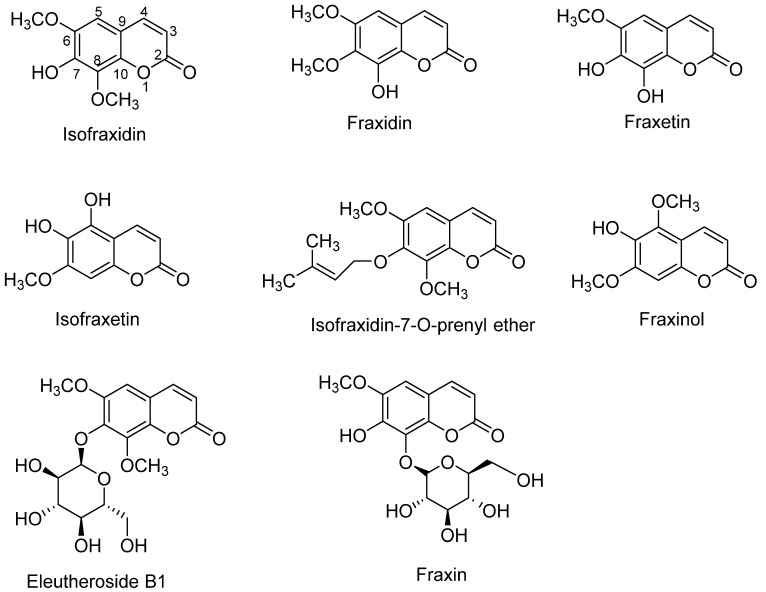
Chemical structure of Isofraxidin (7-hydroxy-6, 8-dimethoxy coumarin) (IF) and other selected coumarin compounds.

The complex pathophysiological mechanisms behind the diseases including cancer, heart failure, and neurodegenerative disorders urge the need for multi-target compounds. Nature is a critical source of multi-target active compounds with low toxicity, low cost, low drug resistance, high efficiency, and overall promising health benefits [1]. Among natural entities, hydroxy coumarins such as 7-hydroxycoumarin [umbelliferone], 6, 7-dihydroxycoumarin [esculetin], 7, 8-dihydroxycoumarin [daphnetin], 7-hydroxy-8-methoxycoumarin [hydrangetin], and 6-methoxy-7-hydroxycoumarin [scopoletin] are major natural compounds with a coumarin-based structure (2*H*-1-benzopyran-2-one) [2,3]. Compelling evidence has presented hydroxy coumarins as promising multi-target agents that have been found in different parts of several plant families including Fabaceae, Rutaceae, Asteraceae, Oleaceae, and Apiaceae [4,5,6]. Besides, owing to their anti-inflammatory [7], anticoagulant [8], antioxidant [9], anticancer [4,10], hepatoprotective [11], antidiabetic [12], neuroprotective [13], anxiolytic [14], antidepressant [15], antibacterial [16], and cardioprotective [17] effects, researchers have focused their attention on the synthesis of coumarins derivatives in addition to their isolation and purification from natural entities [18]. As one of the most prominent hydroxy coumarins, 7-hydroxy-6,8-dimethoxy coumarin [isofraxidin, IF] along with other related lead compounds (Figure 1), such as 8-hydroxy-6,7-dimethoxycoumarin [fraxidin], 7, 8-dihydroxy-6-methoxycoumarin [fraxetin], 5, 6-dihydroxy-7-methoxycoumarin [isofraxetin], 6-hydroxy-5, 7-dimethoxycoumarin [fraxinol], and 7-hydroxy-6-methoxycoumarin 8-glucoside [fraxin] are natural-based multi-targets, isolated from several phytomedicines especially in *Acantopanax, Achillea, Artemisia, and Fraxinus* genera. IF and related compounds attenuate multiple destructive signaling mediators and therapeutic targets in some diseases [19,20,21,22,23,24]. In this regard, IF has shown several biological and pharmacological activities including antioxidant [25], cardioprotective [26], weight loss [27], anti-osteoarthritis [28], antimalarial [29], and neuroprotective [30] effects, with promising anticancer and anti-inflammatory effects. Accordingly, nuclear factor kappa-light-chain-enhancer of activated B cells (NF-κB), toll-like receptors (TLRs), inducible nitric oxide synthase (iNOS), cyclooxygenase-2 (COX-2), tumor necrosis factor-α (TNF-α), inhibitory kappa B (IκBα), mitogen-activated protein kinases (MAPKs), and c-Jun *N*-terminal kinase (c-JNK) are the most targeted pathway for anticancer and anti-inflammatory effects of IF [27,31,32,33,34]. Considering the biological applications of IF, developing the isolation, purification, identification, as well as structure elucidation, synthesis and biosynthesis methods of IF is of great importance. Therefore, several new extraction methods and purification techniques for IF, such as mechanochemically assisted extraction (MCAE) and microwave coupled with high-speed countercurrent chromatography (HSCCC) have been successfully verified [35,36]. On the other hand, owing to the rapid absorption of IF after oral use along with its high penetration to the central nervous system, elucidating its precise pharmacokinetic properties could open new roads to reveal potential biological activities and health benefits of IF [37]. The present study is the first to focus on the synthesis, biosynthesis, isolation, and pharmacokinetic properties of IF. The biological activities, health benefits, and therapeutic potentials of IF in the treatment/prevention of the peripheral and central nervous system diseases are also reviewed.

## 2. Isofraxidin

### 2.1. Natural Sources

Isofraxidin is a hydroxy coumarin compound with IUPAC name of 7-Hydroxy-6, 8-dimethoxy-2*H*-1-benzopyran-2-one (C_11_H_10_O_5_) and molecular weight of 222.19 g/mol (Figure 1). The needles crystalline of IF is predominantly present in a white to yellow color, while it is reported as colorless in some studies, possessing a melting point of 145–150 °C [38,39,40]. The history of isolation and identification of IF dates back to 1937 from the bark of the ash tree (*Fraxinus exelsior,* Oleaceae) by Spath and Jerzmanowska [41,42]. Since then, this compound has been isolated from numerous plant families including Apiaceae, Asteraceae, Oleaceae, Rubiaceae, etc. On the other hand, *Achillea, Artemisia, Fraxinus,* and *Ixora* are among the known genera in which IF has been isolated from root, stem bark, leave, and aerial parts of their species [43,44]. Table 1 shows some herbs and related parts of those families, which IF is separated from them. *Acanthopanax senticosus* and *Eleutherococcus senticosus* (Siberian ginseng, Ciwujia) from the Araliaceae family have been used as medicinal plants in chines since several years ago. IF has been extensively isolated from this plant regarding its pharmacological and therapeutic effects [45]. Also, as shown in Table 1, species of the genus *Artemisia* from the Asteraceae family have the highest abundance in terms of IF isolation. In addition to plant sources, IF has also been found in fungi, as was isolated from an endophytic fungus, *Biscogniauxia cylindrospora* by Wu et al. [46].

### 2.2. Isolation and Purification

Several strategies are used toward the isolation and purification of coumarin compounds. Maceration, reflux, ultrasonic-assisted, and microwave extraction are current methods for the preparation of the crude extract containing IF [29,78,79,80]. These methods of extractions were conducted in the presence of various solvents including medium polar or non-polar solvents such as ethyl acetate (EtOAc) [69], diethyl ether [44], and hot polar ones such as hot ethanol (EtOH) [81], hot methanol (MeOH) [39], and hot deionized water (W) [82]. However, other solvents or a combination of them have also been used [29,74,83].

Xiao et al. purified IF from *Sarcandra glabra* (whole plant) by microwave extraction (2450 MHz, 1000 W) coupled with an HSCCC. In their study, the hexane (Hex)/EtOAc/MeOH/W (1:2:1:2, *v*/*v*/*v*/*v*) was used as the solvent system of HSCCC with the best ability to isolate IF from other compounds in the extract. On the other hand, the most suitable condition of microwave parameters including the solvent type and volume, solid-liquid ratio, related time, and extraction temperature defined as the 20 min usage of 20 mL 60% (*v*/*v*) hydroethanolic solvent (solid-liquid ratio 1:10 mg/mL) under 65 °C for 2 g sample. Then, the obtained extract was directly injected to HSCCC (flow rate: 2 mL/min, resolution speed: 800 rpm, detector wavelength: 344 nm), which rendered 1.2 mg IF from 300 mg dried extract with a purity of about 99%, using high-performance liquid chromatography (HPLC) [36].

**Figure 2:**
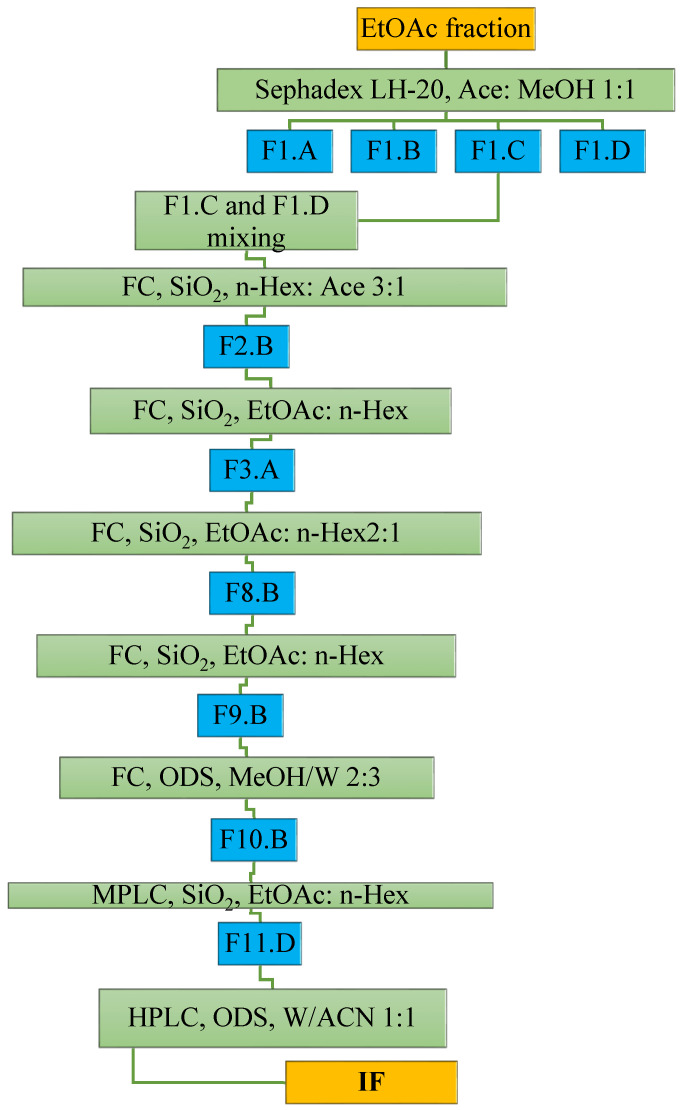
The procedure of *Saposhnikovia* root fractionation to isolate IF. F: fraction, EtOAc: ethyl acetate, Ace: acetone, MeOH: methanol, W: water, FC: flash chromatography, SiO_2_: Silica gel column, *n*-Hex: *n*-hexane, MPLC: medium pressure liquid chromatography, ODS: octadecyl silane column, ACN: acetonitrile, IF: isofraxidin.

In a bioassay-guided purification of IF from the methanolic extract of *Saposhnikovia divaricate* root, the extract was fractionated with EtOAc, *n*-butanol, and W, respectively. The EtOAc fraction was then eluted with acetone: MeOH (1:1) through a Sephadex LH-20 column. The medium eluted fractions were repeatedly purified with the Silica gel column (Hex-EtOAc) and RP-18 (MeOH: W) columns. Final purification with reversed-phase HPLC resulted in 42 mg pure IF with a 40% yield. The summarized fractionation procedure was shown in Figure 2 [74].

The liquid-liquid separation method has also been used in a couple of studies. Tsukamoto et al. purified IF from *fraxinus japonica* bark, with hot MeOH. MeOH extract was partitioned with diethyl ether, *n*-butanol, and chloroform. About 16.7 g diethyl ether extract was fractionated by the Silica gel column eluted with a gradient of EtOAc-chloroform to get 1 mg of pure IF [66]. However, Duc et al. used EtOAc for partitioning of the MeOH extract of *Morus alba* leaves. Then they employed dichloromethane: MeOH and chloroform: EtOAc for the sequence fractionation to purify IF, using column chromatography analyses [69]. Using pretty similar liquid-liquid extraction, Takemoto et al. purified 90 mg of IF from 80 g dried EtOAc extract of *Chloranthus japonicus* root besides using benzene: EtOAc for chromatography [39]. Regarding MCAE as a new extraction method in which the mechanical power such as milling is combined with the chemicals to increase the yield [84], it was also used by Liu et al. to isolate IF from roots and rhizomes of *Eleutherococcus senticosus.* They showed that the MCAE method was more economical with a shorter extraction time. Accordingly, they compared MCAE (Vibration mill and Na_2_CO_3_ in concentration range 0.25–8%, *w*/*w*) with no mechanical extraction (heat-reflux with hydroethanolic solvent 0–100%) and mechanical extraction/vibration mill (WZJ (BFM)-6J) without chemical substance. The results showed that the highest yield of IF (0.482 mg/g) was obtained by mechanochemical extraction at 25 °C in water for 5 min. Superfine sized (*D*95 ≤ 44 µm), 2% *w*/*w* Na_2_CO_3_, 20/1 mL/g as solvent/solid ratio were other optimized conditions [85]. Besides, IF separation was also done using W extraction of *Acanthopanax senticosus* with macroporous resins (HPD100C had best results, % recovery: 93.79%) [83]. In general, semi-polar solvents such as EtOAc are most commonly used for extraction and purification of IF. So, the aforementioned new methods have been successfully used to isolate and purify IF with a high recovery percentage.

### 2.3. Identification and Structure Elucidation

Nowadays, spectroscopic methods including UV-spectroscopy, infrared (IR), nuclear magnetic resonance (^1^H NMR, ^13^C NMR), and mass spectroscopy are used for identification and structure elucidation of natural compounds such as coumarins. Melting point, type, and color of compound crystals are also of the other indicators [86,87]. Heterocyclic molecules such as coumarins have a combination of electronic transitions between π-π* and *n*-π* that is related to the presence of α-pyrone and benzene rings, as well as the carbonyl group. Also, the presence of a strong conjugate system in α, β-unsaturated ketone structure of coumarins has led to a stronger bathochromic shift and higher wavelengths. On the other hand, the presence of groups containing non-bonding electrons and electron donors, such as methoxy and hydroxyl groups in the IF structure, strengthens the existing π system and increases the bathochromic shift relative to the basic structure of coumarins [88,89]. The UV absorption range of coumarins’ basic structure is about 220–310 nm (in EtOH), which this range for IF was reported λ_max_ (in MeOH) 212–228–344 nm [39,70,90,91]. Additionally, the IR spectroscopy of IF showed several prominent absorption spectra (KBr) including 3200–3500 cm^−1^ (the broad peak related to the hydroxyl group (OH^−^), 2900–3000 cm^−1^ (medium, C-H), 1675–1705 cm^−1^ (strong, C=O), 1570–1600 cm^−1^ (medium, C=C aromatic), 1600–1680 cm^−1^ (weak, C=C), and 1030–1300 cm^−1^ (medium, C-O) [92,93]. In another study, the noticeable results of IR spectroscopy of IF isolated from *Sarcandra glabra* were included 3298, 2981, 2941, 1705, 1606, 1574, 1302, 1036 that could be interpreted based on the aforementioned results [94]. The range of the chemical shifts (δ) and coupling constants (*J*) of the ^1^H and ^13^C NMR study of IF has been shown in Table 2. The main reason for the appearance of H-4 in the weaker field than other IF protons is it’s de-shielding due to the conjugated resonance between the double bond C3=C4 and the carbonyl group of the α-pyrone ring. H-5 also exhibits higher δ than H-3 due to the anisotropic effect of the benzene ring and the conjugated resonance with the carbonyl group of the α-pyrone ring. On the other hand, δ_c_ of the IF structure can be justified by the conjugated resonance with the carbonyl group, the magnetic anisotropy of benzene and α-pyrone ring, and the electronegative effect of the oxygen atom on the vicinal carbon [92,95,96,97,98]. Table 2 shows the approximate range of ¹H (500 MHz, CDCl3) and ^13^C NMR (75 MHz, CDCl3) chemical shifts (δ) of IF [92].

**Figure 3:**
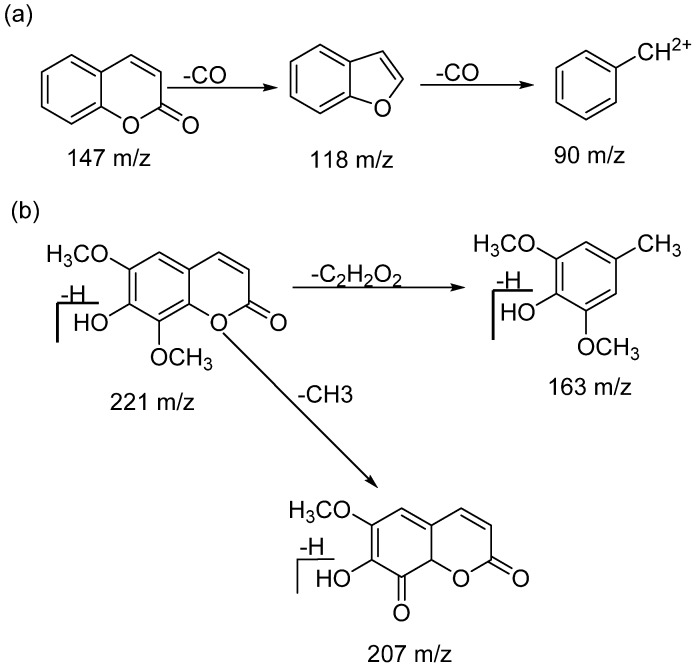
Mass fragmentation pattern of coumarin basic structure (**a**) and IF (**b**).

Mass spectroscopy (MS) study of coumarin compounds has also shown that the losing of C-O moiety is the main pathway of their fragmentation. The results of this fragmentation are producing two molecular ions at 118 *m*/*z* and 90 *m*/*z* [99]. On the other hand, the mass spectroscopy of IF [222, M^+^] showed the fragments at 207 and 179 that indicated the losing of CH_3_ and C-O groups at position C6 and C8. Besides, the appearance of pieces at 194,166, 151(166-CH_3_), and 123 *m*/*z* have been related to fracturing the C-O groups [38,70,100]. Also, another study reported that the IF showed a fragment at 163 *m*/*z* in MS/MS spectrum [30] (Figure 3).

### 2.4. Synthesis and Biosynthesis

Coumarins such as IF were primarily synthesized through different methods including Perkin, Von Pechmann, Knoevenagel, and Wittig reactions [101]. In this regard, Rouessac and Leclerc synthetized IF through a five-step method from a trimethoxylated benzaldehyde with the overall yield of 48%. In their synthesis, Knoevenagel reaction with zinc oxide (140 mmol) was used in the presence of the Meldrum’s acid (14 mmol) to produce the IF with high efficiency and purity. They also used sulfuric acid (in ice bath, 0.5 h) for closing ring of 2,2-dimethyl-5-((4-hydro-2,3,5-trimethoxyphenyl)-methylene)-l,3-dioxane as the precursor of 6,8- dimethoxy-2-oxo-2*H*-chromene-3-carboxylic acid. The last step included converting the obtained coumarinic acid to IF in the presence of powdered copper (36 mg) and heat (300 °C, under N_2_) for 10 min. To purify IF, cyclohexane/EtOAc was used for crystallization [102].

**Scheme 1:**
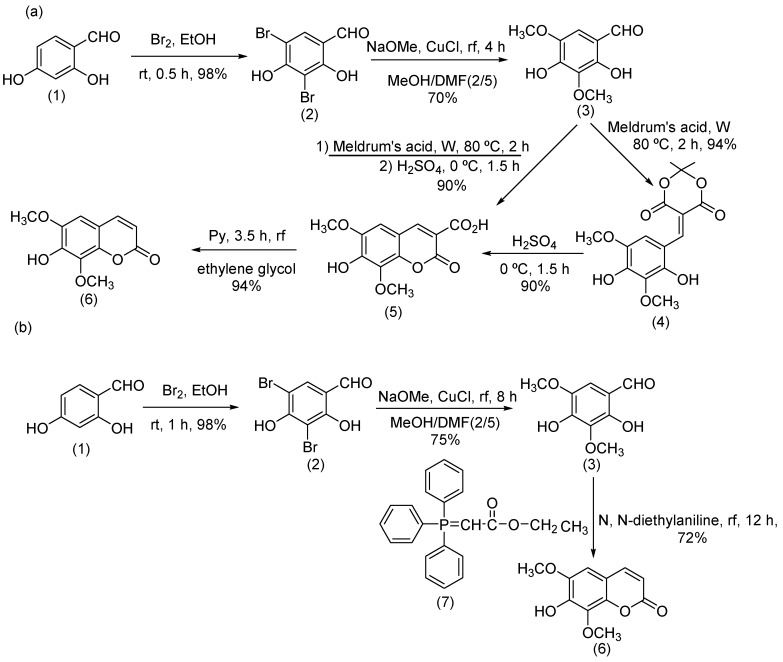
The Synthesis procedure of IF in the modified Knoevenagel (**a**) and Wittig (**b**) methods. (1): 2,4-Dihydroxybenzaldehyde, (2): 3,5-Dibromo-2,4-dihydroxybenzaldehyde, (3): 2,4-Dihydroxy-3,5-dimethoxybenzaldehyde, (4): 5-(2,4-Dihydroxy-3,5-dimethoxybenzylidene)- 2,2-dimethyl-1,3-dioxane-4,6-dione, (5): 7-Hydroxy-6,8-dimethoxy-2-oxo-2H- chromene-3-carboxylicacid, (6): 7-Hydroxy-6,8-dimethoxy-2H-chromen-2-one (Isofraxidin), (7): ethyl (triphenylphosporanylidene) acetate, EtOH: Ethanol, rt: room temperature, MeOH: Methanol, NaOMe: Sodium methoxide, CuCl: Copper chloride, DMF: Dimethylformamide, rf: reflux, W: water, H_2_SO_4_: Sulfuric acid, h: hour, Py: pyridine.

Besides, Silva and co-workers used an environmentally friendly method for the synthesis of IF. Actually, they used a modified one-step Knoevenagel reaction with eliminating the organic co-solvents and the catalyst, where used Meldrum’s acid. Accordingly, they used the 2,4-dihydroxybenzaldehyde (commercially purchased, Scheme 1, compound (**1**)) as a precursor for the synthesis of IF. In their study, the first step was the dibrominating of compound (**1**) (20 mmol dissolved in the 40 mL EtOH) in the presence of bromine (40 mmol) at room temperature (0.5 h) with the yield of 98%. Then, 3,5-dibromo-2,4-dihydroxybenzaldehyde (Scheme 1, compound (**2**)) (40 mmol dissolved in 70 mL of the anhydrous MeOH and anhydrous dimethylformamide at 2:5 ratio) was heated with copper chloride (5 mmol) and sodium methoxide (400 mmol) under reflux at 100 °C (4 h, 70% yield). After that, 1 mmol of the obtained compound (**3**) (2,4-dihydroxy-3,5- dimethoxybenzaldehyde) was dissolved in 2 mL W and was stirred for 2 h at 80 °C after mixing with Meldrum’s acid (1 mmol). Continuously, the attained 5-(2,4-dihydroxy-3,5-dimethoxybenzylidene)-2,2-dimethyl-1,3-dioxane-4,6-dione (Scheme 1, compound (**4**)) was acidified by sulfuric acid (0 °C, 1.5 h, pH = 1) for giving the 7-hydroxy-6,8- dimethoxy-2-oxo-2*H*-chromene-3-carboxylic acid (Scheme 1, compound (**5**)). Also, the compound (**5**) was synthesized by merging the steps of adding Meldrum’s acid and acidification with sulfuric acid. Finally, the IF (Scheme 1, compound (**6**)) was obtained after decarboxylation of compound (**5**) in the presence of pyridine and ethylene glycol (reflux, 3.5 h). IF was extracted (yield 94%) by dichloromethane after acidification of the solution with hydrochloric acid (2 M). The synthesis steps have shown in Scheme 1a [103].

On the other hand, Talakokkula and co-workers used a similar method for the synthesis of IF with some significant modifications, especially in the final step (Scheme 1b). Briefly, they synthesized compound (**2**) from compound (**1**) (commercially purchased) through bromination (0.1 mL, 3.26 mmol, stirred for 1 h). Then compound (**3**) was synthesized from compound (**2**) (2.3 mmol) after stirring and refluxing for 8 h in presence of the copper chloride (0.23 mmol), sodium methoxide (23.3 mmol) and dimethylformamide. In the final step, they used Wittig reaction in the presence of ethyl (triphenylphosporanylidene) acetate (1.42 mmol, Scheme 1b, compound (**7**)) in N, N-diethylaniline (as solvent, 10 mL, reflux 12 h) to convert compound (**3**) to IF (0.32 mmol, 72% yield). The obtained IF was then purified by column chromatography with petroleum ether-EtOAc in the ratio of 3:1 as a solvent system [104].

Wittig reaction was also used to synthesize IF by Gao and co-workers. Accordingly, IF was synthesized after mixing 1 mmol of the compound (**3**) and 1.2 mmol of the compound (**7**) in 1.5 mL of the *N*, *N*-diethyl aniline as a solvent and refluxing under the N_2_ atmosphere for 15 min (yield 57.6%). The obtained brown oil was purified from the other hydroxylated and methoxylated coumarins by column chromatography with petroleum ether-EtOAc as the mobile phase. In their study, the hydroxy group of 4-hydroxy-3,5-dimethoxybenzaldehyde (as a precursor of IF) was protected by pivaloyl chloride and resulted compound (4-formyl-2,6-dimethoxyphenyl pivalate) was iodinated by *N*-iodo-succinimide to increase the efficiency [92].

Regarding the aforementioned synthesis methods, Silva and co-workers used a modified Knoevenagel reaction, with environmental benefits, and a more significant yield (94%) [103].

**Figure 4:**
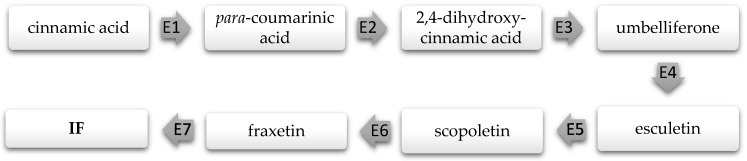
Proposed pathway of the IF biosynthesis. E1: Cinnamic acid 4-hydroxylase, E2: Coumarate/cinnamate/ferulate 2-hydroxylase, E3: Intramolecular lactonization, E4: Esculetin synthase, E5: Methoxy transferases, E6: scopoletin 8-hydroxylase, E7: Coumarin methyltransferase.

The biosynthetic origin of coumarins involves the shikimic acid pathway, which it seems to be an important way for the biosynthesis of the C6-C3 units (phenylpropane derivatives). This pathway has seven enzymatic steps to produce chorismic acid, which is regarded as an essential branching point for the biogenesis of different products. Among these products, phenylalanine can be converted to cinnamic acid under the influence of the enzyme phenylalanine ammonia-lyase [105,106]. Actually, the *ortho*-hydroxylation of the benzene ring of the cinnamic acid, followed by *cis* and *trans* isomerization and lactonization of the hydroxyl cinnamic acid results in the formation of the core structure of coumarins [107,108]. The cytochrome P450 enzymes such as cinnamate 4-hydroxylase, 2-hydroxylase, and 2-oxoglutarate dependent dioxygenase also have a critical role in the hydroxylation of *ortho*-position of the aromatic ring of cinnamates in the biosynthesis of hydroxylated coumarins such as umbelliferone, scopoletin and IF [2,109,110,111]. Additionally, methoxy transferases also have a critical role in the formation of methoxylated coumarins [112]. Although, there is no exclusive report on the biosynthesis of IF, Brown, and co-workers showed that IF has a precursor role in the biosynthesis of 7-*O*-prenyl ether of IF (Figure 1). In their proposed pathway, 4′-hydroxycinnamic acid is converted to umbelliferone, and subsequently esculetin, scopoletin, fraxetin, and IF [113,114]. Based on other studies, in this biosynthesis pathways, cinnamic acid was converted to *para*-coumarinic acid (4′- hydroxycinnamic acid) under the influence of cinnamic acid 4-hydroxylase. Then *para*-coumarinic acid was converted to 2,4-dihydroxy-cinnamic acid by coumarate/cinnamate/ferulate 2-hydroxylase followed by a spontaneous intramolecular lactonization resulted in the formation of umbelliferone. Umbelliferone was, in turn, converted to esculetin by esculetin synthase, followed by the influence of methoxy transferases, which yielded to scopoletin [2,107,115]. In another study, Tsia and co-workers showed that scopoletin 8-hydroxylase converted the scopoletin to fraxetin [116]. On the other hand, Osoba and Roberts reported that coumarin methyltransferase enzymes have critical roles in the biosynthesis of IF. Actually, they showed that IF was biosynthesized after methylation of the fraxetin by coumarin methyltransferases [117]. The summarized of the proposed pathway of IF biosynthesis has been shown in Figure 4.

### 2.5. Pharmacokinetic Studies

The pharmacokinetic parameters of IF as an active compound of *Acanthopanax senticosus* (Ciwujia) was reported by Li et al. In their study, IF showed a rapid absorption in rat plasma after oral administration *Acanthopanax senticosus* extract by liquid-liquid method (using EtOAc). Results showed some pharmacokinetic parameters of IF including the time of reaching to maximum plasma concentration (Tmax), and the half-life (t1/2), which was reported 0.236 h and 4.262 h, respectively. The maximum plasma concentration (Cmax), area under the curve (AUC_0-t_) and AUC_0-infinity_ were also calculated 5.472 µg/mL, 8.162 µg h/mL and 8.942 µgh/mL, respectively [118]. Besides, pharmacokinetic parameters after oral administration of the pure IF (15 mg/kg) was showed that the elimination half-life (t1/2β) of was 7.89 h, indicating the slow rate of plasma clearance of pure IF from rat plasma. The other pharmacokinetic parameters of the same dose of pure IF including t1/2, Cmax, and Tmax, was calculated 0.57 h, 13.80 µg/mL, and 0.23 h, respectively. It revealed different pharmacokinetic behavior of IF in the *Acanthopanax senticosus* extract in comparison to pure IF. On the other hand, the presence of eleutheroside B1 as a precursor of IF in the *Acanthopanax senticosus* extract has led to the release of IF with two-compartments pharmacokinetic models (0–4 h and 4–24 h). Therefore, the AUC of *Acanthopanax senticosus* extract-containing IF and pure IF was markedly different, and its peak was detectable for up to 24 h, while the peak of pure IF disappeared at 15 h [119].

Also, Sun and co-workers used the ultra-performance liquid chromatography coupled with electrospray mass spectrometry for the determination of IF (p.o., 10 mg/kg) and its metabolites in rat plasma. Their study showed that liver plays a critical role in the metabolism of IF and produced two metabolites including 7,8-dihydroxyl-6-methoxy coumarin and 8-hydroxyl-6,7-dimethoxy coumarin [120]. Besides, the high lipophilicity characteristic of IF caused the rapid pass from the blood-brain barrier, possessing the concentration of 292.97 ng/mL IF in the extracellular fluid of rat striatum 5 min after orally used. In their study, the Tmax was calculated 15 min after oral administration of 10 mg/kg and 20 mg/kg of IF for the extracellular fluid of rat corpus striatum. Also, the Cmax and AUC_0-inf_ of 20 mg/kg IF were calculated 899.82 ng/mL and 28059.76 ng min/mL, respectively, dose-dependently [37].

The thermodynamic parameters of IF interaction with human serum albumin and bovine serum albumin showed that hydrophobic interaction has a critical role in related binding [121,122]. Besides, Sun and co-workers showed that IF has pharmacokinetic interactions with related drugs that are metabolized via cytochrome P450 isoenzymes. This is owing to the inhibitory effects of IF on cytochrome P450 enzymes including CYP1A2, CYP3A4, and CYP2E1 [123]. On the other hand, glucuronidation is one of the most important pathways of IF metabolism and two isoforms of human UDP-glucuronosyltransferase (UGT): UGT1A1 and UGT1A9 [124].

## 3. Pharmacological Properties of IF: Biological Activities and Therapeutic Potentials

### 3.1. Anti-Inflammatory Effects

IF is one of the most prominent anti-inflammatory coumarins. Most recently, Su et al. studied the effectiveness of pure IF on reducing inflammation mediators in human nucleus pulpous cells that were induced with interleukin-1β (IL-1β, 1 ng/mL). They showed that IF markedly reduced inflammatory cytokines such as tumor necrosis factor-alpha (TNF-α) and IL-6 at the doses of 10, 20, and 40 µM. Pretreatment of nucleus pulpous cells with IF showed protective effects on their survival against IL-1β. On the other hand, the same concentrations of IF blocked all inflammatory mediators including COX-2, nitric oxide (NO), prostaglandin E2 (PGE2), and iNOS. Besides, the expression of MMP-3 and MMP-13, as well as the NF-κB activation, induced by IL-1β (1 ng/mL), was significantly decreased with IF. In their investigation, all the anti-inflammatory effects of IF were dose depended, and researchers proposed the beneficial effects of pure IF in the intervertebral disc degeneration [125].

The anti-osteoarthritis effects of pure IF were investigated via the inhibition of inflammatory factors in two studies. In their investigations, IF significantly reduced the activity and expression of NO, PGE2, TNF-α, IL-6, COX-2, iNOS, MMP-1, MMP-3, and MMP-13 [28,126]. A disinterring and metalloproteinase with thrombospondin motifs 4 and 5 (ADAMTS-4 and ADAMTS-5) have shown a critical role in cartilage destruction, and their expression is increased in osteoarthritis patients [127]. Pretreatment of human osteoarthritis chondrocytes for 2 h with IF (1, 10, and 50 µM) in medium culture reduced the expression of ADAMTS-4 and ADAMTS-5, induced by IL-1β [126]. Also, lipopolysaccharide (LPS) caused to couple the TLR4 with myeloid differentiation protein-2 (MD-2) and the formation of TLR4/MD-2 complex and stimulated the inflammation in OA patients [128,129]. Pure IF (20 µM) inhibited the formation of TLR4/MD-2 complex induced by LPS (1 µg/mL) with binding to the MD-2 junction located at TLR4, in vitro [28]. On the other hand, Yamazaki and co-workers also suggested IF and syringaresinol for the treatment of osteoarthritis. They reported that syringaresinol more inhibited the pro-inflammatory factors including IL-1β, IL-6, COX-2, and MMP-1 in human synovial sarcoma cells (SW982) through blocking the activity of NF-κB and activating protein-1 (AP-1) [130]. The intraperitoneal injection (i.p.) of 15 mg/kg IF remarkably decreased ear edema and showed analgesic effects in mice induced by xylene and acetic acid, respectively. Also, in the formalin-induced pain test, it was demonstrated that IF (15 mg/kg) reduced pain and the edema induced by carrageenan. IF (10 mg/kg) significantly decreased pain and edema through decreasing the expression of TNF-α, and extracellular signal-regulated protein kinases 1 and 2 (ERK1/2) [32].

Liu and co-workers also evaluated the in vivo anti-inflammatory effects of pure IF. They showed that IF increased the survival rate of mice at all doses being assessed (1, 5, and 15 mg/kg, i.p.) against LPS (1 mg/kg, i.p.). The study found that IF significantly decreased the serum levels of IL-6 at a dose of 15 mg/kg against LPS pretreatment. IF also suppressed the release of NF-κB p65 from mice liver and the increased expression of TNF-α, which was stimulated with LPS. IF also inhibited LPS-induced weight gain and edema of the lung and liver but did not affect the heart [33]. Altogether, the anti-inflammatory effects of IF could be used to combat various diseases. Accordingly, IF has shown an excellent anti-inflammatory activity through the regulation of several signaling pathways including NO, PGE2, TNF-α, IL-6, ADAMTS, COX-2, iNOS, MMPs, etc. Therefore, it can be introduced as a potential molecule in the treatment of inflammatory diseases such as osteoarthritis and pain.

### 3.2. Antioxidant Activity

Wang and co-workers showed that IF separated from *Chimonanthus salicifolius*, scavenged free radical 2,2-diphenyl-1-picrylhydrazyl (DPPH) at EC50 of 78.01 µM, while the EC50 for 7-hydroxy-6,8-dimethoxy-3-(6-methoxy-2-oxochromen-3-yl)oxychromen-2-one, 3,3′-biisofraxidin and scopoletin were 96.11 µM, 49.87 µM and 65.89, respectively [40]. The antioxidant activity of IF, isolated from the roots of *Anneslea fragransfor*, scavenging 2,2′-azino-bis(3-ethylbenzothiazoline-6-sulfonic acid) and DPPH with IC50 of 77.3 ± 3.6 µg/mL and 51.6 ± 2.2 µg/mL was stronger than scopoletin [25]. Besides, the biosynthesis of IF in leaves of *Fraxinus excelsior* was significantly increased when exposed to oxidative stress with ozone [131]. However, the antioxidative effects of IF are not yet noticed enough and require further in vitro and in vivo studies.

### 3.3. Anticancer and Cytotoxic Activities

The cytotoxic effects of IF (commercially purchased) were reported in several studies. Zhang and co-workers showed that IF possesses apoptosis-inducing activities against human lung cancer cell line A549. In their research, the IC50 of IF antiproliferative effect was reported 75.16 ± 3.42 μM for 72 h treatment in a dose and time-dependent manner. Additionally, the results of the flow-cytometer assay revealed that IF with an IC50 value of 40 µM caused an arrest in phase S of the cell cycle. On the other hand, it was found that the expression ratio of Bax/Bcl-2, as an apoptotic index, was increased along with a decrease in the rate of the migratory/invasive cell, as metastatic ability following IF administration. The IC50 value of IF for cytotoxic effect on normal lung epithelial cells calculated 85.32 ± 2.34 μM after 48 h treatment, confirming the low side effects of IF on normal cells (IC50 = 42.71 ± 4.05, 48 h). The in vivo part of their study on IF (5 mg/kg, i.p., 21 days) indicated a significant anti-lung cancer activity of IF in comparison with cisplatin as a positive control. IF (commercially purchased) decreased the weight and volume of the tumors. Also, IF (10, 20, and 40 µM) inhibited the expression of epidermal growth factor receptor (EGFR) through which caused an interfere with other apoptotic signaling pathways [132].

In another study, the cytotoxic effects of IF on human colon cancer cell line (HT-29) and SW-480 as two colorectal cancer cell lines, and related signaling pathways were investigated. IF showed antiproliferation effects with IC50 of 40 µM and 80 µM on HT-29 and SW-480 in 24 h treatment, respectively. This effect was exerted via the inhibition of Akt as a critical kinase involved in the survival and proliferation of cancer cells. IF also caused the accumulation in necrotic cells and changed the morphological characters of cancer cell lines in a time and concentration-dependent manner. The results of their study revealed significant apoptotic effects of IF through increasing caspase-9, Bax/Bcl-2, and caspase-3 [133].

The results of an in vitro study revealed the signaling pathways involved in metastatic and invasive inhibitory effects of IF (30 and 100 µM), purchased commercially, in human hepatoma cells (HuH-7 and HepG2) including blocking the expression of MMP-7 induced with 12-*O*-tetradecanoylphorbol-13-acetate (80 nM), while IF did not show cytotoxic effects on hepatoma cells including HepG2, HLE, and HuH-7 [134]. The pure IF, isolated from *Micrandera elata,* also showed cytotoxic activities against murine leukemia (P388, ED50 = 1.7 µg/mL), with no effects on human mouth epidermal carcinoma (KB, ED50 > 100 µg/mL) [70]. Also, Steinberg and co-workers reported that IF, isolated from *Ruyschia phylladenia*, had no cytotoxicity on cancer cell lines including two lines of human breast adenocarcinoma (MCF-7, MDA-MB-231) and human bladder carcinoma cells with low activity (IC50 = 51.9 µg/mL) on BALB/c mouse macrophages [29].

On the other hand, the chemoprotective effects of pure IF were approved in several investigations. Li and co-workers reported the radioprotective results of IF. In their study, lymphoma cell lines including HL60, Molt-4, and U937, were pre-treated with IF for 1 h, then the cells were exposed to X-rays (10 Gy). The results showed that IF reduced the hydroxyl radical and reactive oxygen species (ROS) in the intercellular media of U937. IF (500 µM, 1 h) also blocked one of the mitochondrial pathways, thereby activated apoptosis, via a significant reduction in the phosphorylation of MAPKs members including c-JNK and p38 after exposing U937 cell line to X-rays (10 Gy). The results of DNA fragmentation test and cell counting test also revealed the protective and antiapoptotic effects of IF (500 µM, 1 h) for other leukemia cell lines including HL60 and Molt-4 against X-rays [34].

As a main compound of *Acanthopanax senticosus*, IF extracted from decoction of the mixture of *Acanthopanax senticosus* and *Ligustrum lucidum* possessing a more significant protective effect against bone marrow suppression induced by chemotherapy than either of these herbs alone. In their study, the solubility of the effective constituents of both herbs, such as IF and specnuezhenide, increased with concomitant decocting, which is directly related to their protective effects against cyclophosphamide-induced bone marrow suppression as a chemotherapy drug [135]. In general, the above investigations showed that IF has hopeful anticancer and cytotoxic effects against several types of cancers including lung cancer, breast cancer, colon cancer, leukemia, and hepatoma, through the inhibitory effect on apoptosis, metastasis, and invasive signaling pathways. Nevertheless, more in vitro and in vivo studies are recommended.

### 3.4. Cardioprotective Effects

IF has also shown anti-hypertension effects via inhibiting the activity of angiotensin I converting enzyme (ACE). Cho and co-workers, in two separate studies on *Artemisia scoparia*, demonstrated the effectiveness of purified IF (1 mM) on the blockade of ACE activity (69.0%). Also IF isolated during the EtOAc and chloroform extraction of aerial parts of *Artemisia scoparia,* showed ACE inhibitory effects at 26.1% and 30.6%, respectively [59,136]. On the other hand, Simaratanamongkol and co-workers demonstrated the synergistic effects of IF isolated from *Apium graveolens,* with junipediol A on the inhibition of ACE. The 300 µg/mL of IF, exerted no effect on the inhibition ACE, while 250 µM and 500 µM of Junipediol A exerted 65.9% and 80.7% inhibitory effects on ACE, respectively [52]. On the other hand, in a recent study, IF was also introduced as a cardioprotective agent against myocardial infarction through inhibiting NOD-like receptor family, pyrin domain containing 3. So, these results and those of other studies stated that IF could be a promising cardioprotective agent [26]. However, there is not yet more information on the cardioprotective effects of IF.

### 3.5. Neuroprotective Effects

Siberian ginseng (*Acanthopanax senticosus* or *Eleutherococcus senticosus*) was widely used as neurotonic in Chinese folk medicine [45]. The *n*-butanol and EtOAc fractions of Siberian ginseng methanolic extract led into the coumarins purification including IF, scopoletin, and eleutheroside B2 which all have shown anti-Alzheimer effects via applying protective effects on axons atrophies and dendrites against amyloid β (25–35, 10 µM). Pure IF at 1 µM and 10 µM concentrations also showed remarkable effects on the lengths of axons and dendrites following a 4-day treatment [137,138]. Also, the monoamine oxidase B as the enzyme degeneration of nerve cells, involved in some neurological diseases including Alzheimer’s disease was inhibited by pure IF, isolated from *Acanthopanax senticosus*, with a binding degree of 7.41% that had been determined by UPLC/MS/MS [30]. Considering these neuroprotective effects of IF, conducting more extensive studies on other aspects of this effect and involved pharmacological mechanisms could be helpful in introducing IF as a new drug molecule in the treatment of neurological diseases.

### 3.6. Miscellaneous Effects

Several other biological activities and health benefits have been known for IF. Accordingly, IF (20 mg/kg/day) remarkably reduced weight gain of mice against a high-fat diet (HFD) including 60% fat, 20% proteins, and 20% carbohydrates. In their investigation, IF (commercially purchased) was shown to reduce the serum and liver level of cholesterol versus HFD group. Also, IF (30 mg/kg/day) significantly reduced the serum glucose and alanine aminotransferase in HFD group with no significant effects on aspartate aminotransferase. On the other hand, IF improved fatty acid metabolism in HFD group with an increase in the activity of adenosine monophosphate-activated protein kinase as a cell-protective enzyme versus the energy deficiency stress [139]. Pure IF also improved the phosphorylation of acetyl-CoA carboxylase and inhibited the expression of fatty acid synthase and hydroxyl-3-methylglutaryl coenzyme A reductase [27]. Yim et al. reported a hyperpigmentation activity for IF (commercially purchased) and IF 7-*O*-(6′-*O*-*p*-coumaroyl)-β-glucopyranoside (IFG), isolated from *Artemisia capillaris.* Based on their study, IF (25 µM) and IFG (25 µM) significantly increased melanin content in murine melanoma cells (B16F10). The IFG (25 µM) notably increased the expression of the microphthalmia-associated transcription factor, and tyrosinase as critical proteins in survival and migration of melanocytes [140], more than IF (25 µM). The in vivo part of their study showed that the melanocyte size of zebra fish larva and their tyrosinase activity was increased after treatment with IF (12.5 and 25 µM) and IFG (12.5 and 25 µM) [93]. The EtOAc fraction of *Artemisia capillaris* and purified IF reduced the platelet aggregation through the inhibition of arachidonic acid and collagen activity as precursors of platelet aggregation. The pure IF (0.3 mg/mL and 0.22 mg/mL) reduced the arachidonic acid and collagen activity at 100% and 54%, respectively and the EtOAc fraction (10 µg/mL) showed these inhibitory effects at 81% and 70%, respectively [55]. Purified IF from *Artemisia abrotanum* also showed an antimalarial effect against *Plasmodiurn falciparum* (IC50 = 7.95 µg/mL) [57], and antileishmanial activity against promastigotes of *Leishmania amazonensis* with IC50 of 11.1 µg/mL [29]. Although IF isolated from *sarcandra glabra* had no effects on *Staphylococcus aureus* [141], hopeful analgesic effects were reported for IF, isolated from *Saposhnikovia divaricata* Root, by Okuyama and co-workers. In their study, IF significantly reduced pain at a dose of 3 mg/kg in mice (using writhing test). However, its methanolic crud extract also showed analgesic effects at 2 g/kg [74].

**Figure 5:**
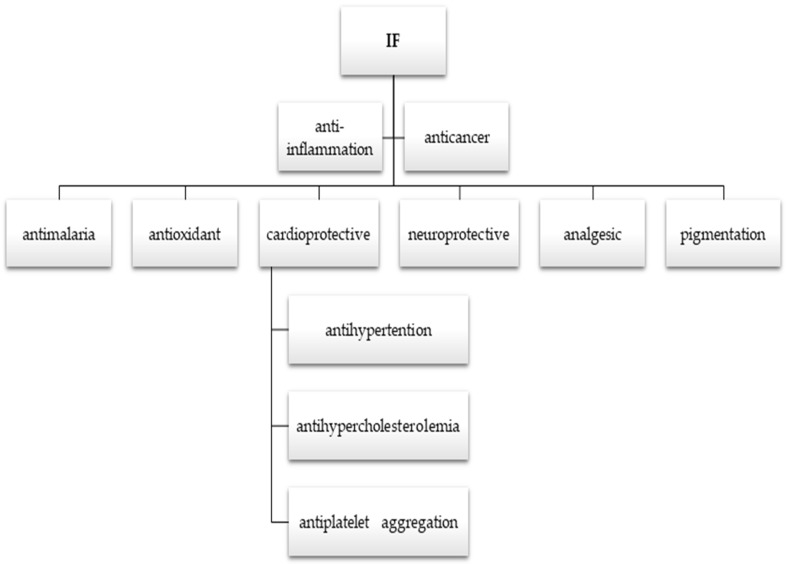
Pharmacological effects of IF. IF: isofraxidin.

Due to the fact that IF shows several biological and pharmacological effects in vitro and in vivo, the molecule can be introduced as a promising drug with multi-therapeutic targets. The summarized pharmacological effects of IF have been shown in Figure 5.

## 4. Conclusions

As promising lead compounds, coumarins have revealed several biological activities and health benefits in combating diseases. Accordingly, these compounds are of great interest owing to their well-known pharmacological effects, which attract special attention for further studies on their pharmacokinetic and chemical properties. IF, as a hydroxy coumarin, is widely found in the plant kingdom, especially the Asteraceae family. EtOAc has been used as a semi-polar solvent more than other solvents regarding a high yield isolation rate. On the other hand, spectroscopic methods such as NMR, UV, and Mass spectroscopy have also been successfully used to identify the IF structure. From the synthesis point of view, despite several synthesis methods for IF, the main path of IF biosynthesis in plants has not yet been revealed. Whereas amongst all the methods used for coumarin synthesis, Knoevenagel reaction was found to be the most applicable method for IF synthesis. Besides, the multi-step enzymatic synthesis pathway was also proposed for the biosynthesis of IF from cinnamic acid, mediated by the impressive role of coumarin methyltransferase enzymes. IF is a small molecular coumarin with hopeful anti-inflammatory effects, leading to potential health benefits including anticancer, antioxidant, neuroprotective, cardioprotective, antihypercholesterolemia, antimalarial, antiplatelet aggregation, and analgesic effects. From the mechanistic point of view, IF attenuated several signaling pathways, thereby play a critical role in disease progression. Among these signaling mediators, NO, PGE2, TNF-α, IL-6, ADAMTS-4 and ADAMTS-5, COX-2, iNOS, TLR4, MMPs, EGFR, Bax/Bcl-2, MAPKs are of more importance. Also, IF affected the activity of various enzymes such as ACE, monoamine oxidase B, acetyl-CoA carboxylase, hydroxyl-3-methylglutaryl coenzyme A reductase and tyrosinase involved in the pathogenesis of some disorders. Owing to the complex pathophysiological mechanisms of the aforementioned diseases, IF could be introduced as a potent multi-target agent, existing in herbal sources, with multiple methods of isolation/purification/synthesis, unique pharmacokinetic profile, and low levels of side effects could be of great importance. Therefore, IF could be introduced as a multi-target agent.

In the present review, all the biological activities, health benefits, and pharmacological mechanisms of action of IF, along with the synthesis, biosynthesis, isolation, and purification methods, have been revealed. Further knowledge of the IF mechanisms and chemical properties will help to elucidate novel clinical application against several diseases. A future area of research should include extensive in vitro and in vivo studies to pave the roads for well-controlled clinical trials.

Such studies will help to provide the more potential uses of coumarins in the prevention, management, and treatment of numerous diseases.

## Figures and Tables

**Table 1:** The plants, their family, and parts that IF is isolated from them.

Genus and Species	Family	Part(s)	Ref.
*Acantopanax senticosus (Eleutherococcus senticosus)*	Araliaceae	root and rhizome	[47]
*Achillea filipendulina*	Asteraceae	root	[22]
*Achillea clavennae*	Asteraceae	aerial parts	[48]
*Achillea depressa*	Asteraceae	root	[44]
*Achillea ochroleuca*	Asteraceae	root	[49]
*Anemone tomentosa*	Ranunculaceae	root	[50]
*Angelica dahurica*	Apiaceae	root	[51]
*Anneslea fragrans*	Theaceae	root	[25]
*Apium graveolens*	Apiaceae	whole plant	[52]
*Apocynum venetum*	Apocynaceae	leaves	[53]
*Arabidopsis thaliana*	Brassicaceae	root	[54]
*Artemisia alba*	Asteraceae	aerial parts	[20]
*Artemisia capillaris*	Asteraceae	aerial parts	[55]
*Artemisia incisa*	Asteraceae	aerial parts	[56]
*Artemisia abrotanum*	Asteraceae	aerial parts	[57]
*Artemisia keiskeana*	Asteraceae	whole plant	[58]
*Artemisia scoparia*	Asteraceae	aerial parts	[59]
*Artemisia selengensis*	Asteraceae	aerial parts	[60]
*Impatiens balsamina*	Balsaminaceae	root	[61]
*Chimonanthus salicifolius*	Calycanthaceae	aerial parts	[62]
*Chloranthus anhuiensis*	Chlorantaceae	whole plant	[63]
*Chloranthus japonicus*	Chlorantaceae	root	[39]
*Daboecia cantabrica*	Ericaceae	flower	[21]
*Erica spp*	Ericaceae	flower	[21]
*Euphorbia lagascae*	Euphorbiaceae	seed	[64]
*Fraxinus exelsior*	Oleaceae	bark	[65]
*Fraxinus japonicus*	Oleaceae	bark	[66]
*Fraxinus ornus*	Oleaceae	bark, flowers, leaves	[67]
*Greenea corymbose*	Rubiaceae	stem bark, leaves	[43]
*Greenea montana*	Rubiaceae	stem bark, root bark, leaves	[43]
*Ixora cibdela*	Rubiaceae	stem bark, root bark	[68]
*Ixora javanica*	Rubiaceae	stem bark, root bark	[43]
*Morus alba*	Moraceae	leaves	[69]
*Micrandra elata*	Euphorbiaceae	twig	[70]
*Pelargonium reniforme*	Geraniaceae	root	[71]
*Peucedanum praeruptorum*	Apiaceae	root	[72]
*Rhododendron rubiginosum*	Ericaceae	leaves	[73]
*Ruyschia phylladenia*	Marcgraviaceae	bark	[29]
*Saposhnikovia divaricata*	Apiaceae	root	[74]
*Sarcandra glabra*	Chloranthaceae	whole plant	[75]
*Sapium discolor*	Euphorbiaceae	stem, leaves	[76]
*Solanum indicum*	Solanaceae	seed	[77]

**Table 2:** The approximate range of ¹H and ^13^C NMR chemical shifts (δ) of IF.

Position	δ_H_	δ_c_
1	-	-
2	-	160.60
3	6.29 (1H, *d*, *J* = 9.5 Hz)	112.50
4	7.60 (1H, *d*, *J* = 9.5 Hz)	145.30
5	6.16 (1H, s)	105.00
6	-	146.10
7	-	143.50
8	-	135.20
9	-	144.50
10	-	110.70
6-OCH_3_	3.95 (3H, s)	56.60
8-OCH_3_	4.10 (3H, s)	61.20

## Abbreviations

ACE	angiotensin converting enzyme
ADAMTS	thrombospondin motifs
AUC	area under the curve
B16F10	a melanin content in murine melanoma cell line
c-JNK	c-Jun N-terminal kinase
Cmax	maximum plasma concentration
COX-2	cyclooxygenase-2
CYP	cytochrome P450
DPPH	2, 2-diphenyl-1-picrylhydrazyl
ERK1/2	extracellular signal-regulated protein kinases 1 and 2
EtOAc	ethyl acetate
EtOH	ethanol
HepG2	a human hepatoma cell line
Hex	hexane
HFD	high-fat diet
HL60	lymphoma cell lines
HLE	a human hepatoma cell line
HPLC	high-performance liquid chromatography
HSCCC	high-speed countercurrent chromatography
HT-29	a human colon cancer cell line
HuH-7	a human hepatoma cell
i.p.	Intraperitoneal injection
IF	Isofraxidin, IFG
IFG	IF 7-*O*-(6′-*O*-p-coumaroyl)-β-glucopyranoside
IL-1β	interleukin-1β
iNOS	inducible nitric oxide synthase
IR	infrared spectroscopy
IκBα	inhibitory kappa B
KB	a human mouth epidermal carcinoma cell line
LPS	lipopolysaccharide
MAPKs	mitogen-activated protein kinases
MCAE	mechanochemically assisted extraction
MCF-7	a human breast adenocarcinoma cell line
MD-2	myeloid differentiation protein-2
MDA-MB-231	a human breast adenocarcinoma cell line
MeOH	methanol
MMPs	matrix metalloproteinases
Molt-4	a lymphoma cell line
MS	Mass spectroscopy
NF-κB	nuclear factor kappa B
NMR	nuclear magnetic resonance
NO	nitric oxide
P388	a murine leukemia cell line
PGE2	prostaglandin E2
ROS	reactive oxygen species
SW982	human synovial sarcoma cells
t1/2	half-life
TLRs	toll-like receptors
Tmax	time of reaching to maximum plasma concentration
TNF-α	tumor necrosis factor-α
U937	a lymphoma cell line
UGT	UDP-glucuronosyltransferas
W	water
TLRs	toll-like receptors
Tmax	time of reaching to maximum plasma concentration
TNF-α	tumor necrosis factor-α
U937	a lymphoma cell line
UGT	UDP-glucuronosyltransferas
W	water
